# (New) Realist Social Cognition

**DOI:** 10.3389/fnhum.2020.00337

**Published:** 2020-09-10

**Authors:** Nicolás Araneda Hinrichs

**Affiliations:** ^1^Laboratorio de Psicolingüística, Universidad de Concepción, Concepción, Chile; ^2^Institut für Angewandte Linguistik und Translatologie, Universität Leipzig, Leipzig, Germany

**Keywords:** social cognition, sensorimotor coupling, new realism, ontology, epistemology, 4E-cognition

## Introduction

Low-level descriptions of interaction dynamics have been canonically approached by cognitive neuroscience through a representation-oriented and inference-based perspective, leading to a stable paradigmatic *plateau*, that no longer allows further construction of a completely coherent semiotic framework capable of accounting for currently unobserved characteristics of social cognition, which is forcibly situated and mostly occurs in interaction. Social contexts are saturated with information that remains invisibilized because of the use of mutually incommensurable conceptual metaphors throughout contemporary scientific discursive practices, despite the embodied turn led by 4E Cognition. A new turn toward realist ontology and epistemology is thus rendered as necessary to inform the gaps within cognitive neuroscience and ground its currently unfulfilled interdisciplinarity. Examples are drawn from research on language to make the case for each argument.

Trending cognitive neuroscience performs low-level descriptions of individual or group interactions by the use of state of the art techniques and methodologies. These observations can be defined as being close to the material niveau of the structure and functioning of our organism as a biological entity. Conscious processes like states of emotion, perception or belief formation –all of which motivate human behavior– transcend the reach of this scope, nonetheless. Thus, the general claims about these epistemic constructs, as a whole, should be more qualified.

For instance, psyscholinguistics used to be quite English based and postulated general principles of which later turned to be proven that they were not replicable with other languages. Psychophysical cues in language processing need to be redefined epistemologically from a new materialistic perspective, in order to account for group learning and social transmission of knowledge. Theory of embodiment has provided the starting point for such a cultural approach of cognition, as it has been summarized by Storch and Tschacher (2014): “The social environment affects the embodied mind” (“Die soziale Umwelt beeinflusst den verkörperten Geist”).

## Knowing Knowledge

### Cognitive Canon

Traditional cognitive theory is based mainly around symbolic manipulation, a process that consists of an analysis of incoming information that is fed into a processor, a unit that produces an outgoing result. Operations that mediate that process behave according to syntactic rules, through which this processor finds a semantic meaning.

This point of view, used as a model in linguistic research, has been widely spread, generating as a by-product its usage as a knowledge model of the human body in a generalized fashion (e.g., sensory information considered as input data), regarding ontogenic development and interaction. As has been ascertained so far (Barsalou, 1999; Meteyard et al., 2012; Urrutia and de Vega, 2012), physiology that supports language processing in human beings resists itself to be reduced to the notion of a mere processor; this is a major challenge for the development of artificial intelligence, deep learning and brain-to-computer interfaces. The canonic focus of symbolism, in its analysis, on the processor in regards to its structure –assuming entities are alike at an internal level–, has downplayed the phenomenal content created by the relation of an entity with their rather external counterpart.

Completely detached and exclusively extrospective perspectives have been rendered banal since the emergence of situated and intersubjective based theories, such as 4E-Cognition (Newen, 2018), particularly regarding sensorimotor coupling with sociocultural contexts, which are saturated with unobserved characteristics of interaction, that need to be accounted for within a mechanistic framework (De Jaegher and Di Paolo, 2012; Rojas-Líbano and Parada, 2020).

A promising avenue –within research on the relevance of both sensory-motor information (Wilson, 2002; Gallese, 2007; Shapiro, 2011) and the experiential context of their process of perception– has been opened by the corpus of theories of embodied, embedded, extended and enactive cognition (Varela et al., 2017), which challenged the assumption that nervous systems evolved for abstract thought (in terms of mere throughput processing) and rather did for the adaptive control of action (Semin and Smith, 2007); therefore, conceptual structure ought to be grounded in an experiential foundation specific to the sensory-motor system. However, as Eliasmith (2003) points out, research insight is still intertwined with metaphors specific for researchers' methodologies at the best, and I would argue that, at the worst, it usually remains at the level of object-oriented ontologies.

### 4E Cognition Explanations as Incommensurable Conceptual Metaphors

Philosophical bias, in the context of contemporary cognitive neuroscience, can be explained as the fundamental assumptions made between ontology (what is), epistemology (what can be known) and practical norms (how science should be practice regarding operative/operational concepts such as causality, probability and complexity, while following ideals like objectivity, reliability, validity, coherence, transparency and rationality). As Andersen et al. (2019) state, these “(…) count as biases because they skew the development of hypotheses, the design of experiments, the evaluation of evidence, and the interpretation of results in specific directions,” although “Sometimes these assumptions are chosen deliberately and explicitly by the scientist, and used as auxiliary premises for theoretical purposes.”

Furthermore, as Craver (2014) points out: “Not all of the facts in an ontic explanation are salient in a given explanatory context, and for the purposes of communication, it is often necessary to abstract, idealize, and fudge to represent and communicate which ontic structures cause, constitute, or otherwise are responsible for such phenomena.”

It follows that mutually incommensurable (Kuhn, 1962; Feyerabend, 1970; Popper, 1996) conceptual metaphors (Lakoff and Johnson, 1980), although intrinsic to a phase of paradigmatic stability in scientific cycles of knowledge production, are also symptomatic of a lack of a completely coherent semiotic framework (Proni, 2015) that could account for currently unobserved characteristics of interaction, which saturate social contexts, remaining invisibilized because of these discursive practices: “(…) The linguistic entities that are called “explanations” are statements reporting the actual explanation. Explanations, in this (ontic) view, are fully objective and (…) no epistemically relativized (…)” (Salmon, 1989).

Models of cognition that have been informed by the 4E-Cognition (Wilson, 2002; Glenberg and Kaschak, 2003) epistemologies have failed regarding the conveyance of higher cognitive states and, even more so, social shared meaning and individual/group learning. Indeed, Zlatev (2007) has said regarding embodiment that “There are, however, three major unresolved issues within the current embodied turn in the sciences of the mind” and at least six within the language sciences (Ostarek and Huettig, 2019). The first was mentioned in passing already: there is not one but many different meanings behind the term embodiment, both between and within fields, and the corresponding theories are in general not compatible (Ziemke, 2003). There is no uniform concept of representation within “embodied cognition.”

The central issue with the purely symbolic perspective has thus not been resolved through the embodied turn, as already put forward by Brette (2019): representing is not some kind of register or data structure that we use, but something we do, as “Items, memory, data, structure, etc. can do nothing relevant except influence process flow, and those influences can, in principle, always be built directly into the process organization” (Erdin and Bickhard, 2018).

## Is a Science of Social Cognition Conceivable?

### Perspectivalness

Social cognition demands the exploration of concepts like interiority and intersubjectivity, which have been held in distance from the possibility of being studied in an interactive way and primarily regarded as a mere contextual descriptors for individual mechanisms. Indeed, Frith (2008) has expressed that “mainly third-person aspects of social-cognitive processes have been considered” so far, even though, as Krakauer et al. (2017) have pointed out: “many have argued for the importance of second-person, participatory capabilities.” They have gone as far as to claim that “Insofar as the goal of a neuroscience research question is to explain some behavior, be it a phenomenon form vision, communication, motor control, navigation, language, memory, or decision making, the behavioral research must be considered, for the most part, epistemologically *prior*.”

De Jaegher et al. (2010) had already argued that “the role of interactive and individual elements in social cognition must be systematically re-evaluated” although they concede, “that social cognition may occur in the absence of interaction.”

To probe the access to others' intentions requires escaping an essentialist and universalizing model of theory of mind. Linguists hold that a child cannot proficiently learn to speak without this capacity (Robbins and Rumsey, 2008). Pauen (2012) suggests that knowing this “perspectivalness” directly enhances the ability to take the second-person perspective, which would essentially allow for epistemic replication to take place. Goldman's (2006) simulation theory had already posited as the central problem of imagining another mind's subjective experience the actual capacity for proper categorization of contextual information.

### Agency

An analogous historical case to take into consideration is Dual Inheritance Theory (also known as gene-culture co-evolution), which effectively broadened the scope of what ought to be considered fit for description regarding the interplay of human physiology and cognition. Nonetheless, following the complexity in the notion of agency –as they put it: “Control is delegated to a system of poorly understood internal drives and rewards that direct the activity of the individual” (Cavalli-Sforza and Feldman, 1981)—DIT was limited to treating subjects simply as self-interested machines.

Nowadays, it is possible to account for several of the biases that undergo social learning and knowledge transmission, and thus attempt to quantify the chance over time of aspects of cognition within a mechanistic framework. This allows us to focus, for instance, on usage frequencies [i.e., regularizations (Reali and Griffiths, 2010)] of the “more richly structured” (Lieberman et al., 2007) aspects of language and to inquire if models of neutral selection can account for these behaviors. This could inform theories of cognition across all levels of information-processing systems (Marr, 1982; Pylyshyn, 1984), a prevailing need for which, according to Newberry et al. (2017) there is a consensus among several disciplines (i.e., neuroscience, artificial intelligence, linguistics, philosophy, psychology and anthropology).

Even if one were to continue tackling dimensions that involve representational content, there is an emerging claim within cognitive science of language that semantic composition is the primary structural selection factor over syntactic processing (Blank et al., 2016) and that there is a need for realistic models of what may have selected for their representations (Hauser et al., 2014).

## Positivity and Possibilities of Realism

The epistemic question of how knowledge is being generated and how this is influencing the research results thus arises. Lende and Downey (2012) propose a holistic approach to improve onto this practice: by further strengthening the way we examine the relationship between recollection of objective data on changes in brain activity and the engagement of culture and individuals simultaneously. So called neuroanthropology places the brain at the center of discussions about human nature, following that “the nervous system is our most cultural organ.” It emphasizes the interaction between the sociocultural milieu and its contingent sensory environment at the material level (i.e., in terms of brain percepts). Anthropology has long made the effort to posit the exploration of Self and Otherness within the scope of the cognitive sciences. Likewise, topics such as the representational requirements of cognition in their relation to the dynamic, circular and distributed causal structure of the brain have not been studied through second-person perspective or ethnographic methods yet, but have been limited to be described by the use of questionnaires at the most.

Within a world of causality, Mead (1962) concisely referred to affordances (Gibson, 1977) by commenting on their potentiality: “The chair invites us to sit down.” Thus, they contribute to the emergence of meaning, since the response to the aforementioned invitation does not depend on cognitive representations alone but they come into play “through particular actions and projects of the subjective selves of the sentient entities” (Keane, 2013). These are central concepts of current robotics, artificial intelligence and information architecture upon which the ethnographic method has to shed some light; this possibility needs to be acknowledged for scientific advancement.

The conveyance of New Realism entails this legitimization. As we have learned specifically from linguistics and more broadly from 4E-Cognition that all structure is social in two ways: it exists through construction and acquires meaning through interpretation. Szwedek (2011) referred to “the ultimate source domain” –the physical– that needs to be cross-ontically mapped before any further higher cognitive metaphorization occurs, as “(…) before any entity can be assigned structure or orientation, it must be objectified first.” It follows that we should learn that explorations on social cognition stemming from discursive practices are not entirely materially unobservable and are actually filled with relevant information –social cognitive affordances– which current working metaphors are not able to represent.

## Contingent Cognitive Constellations

We have described the issue of interaction dynamics having been approached to the point of conceptual saturation by cognitive neuroscience, by mainly making use of representation-oriented and inference-based perspectives. The point of overflow has been reached: current working metaphors within contemporary scientific discursive practices, even though informed through 4E Cognition, no longer address the contingency of sociocultural interaction. Nonetheless, embodiment and situationism themselves pointed toward information pervasively present throughout social contexts which still remains invisibilized and thus requires an ontic and epistemic turn to be accounted for.

Following De Lauretis (2004), who referred to theory as being invested in figuring out the now—i.e., the enigma of the world and argued in favor of theoretical inquiry by stating that “(…) thinking, however abstract, originates in an embodied subjectivity, at once over determined and permeable to contingent events,” this invitation to a new turn toward realism is the attempt to gain specific insight into a contingent social cognition by way of observing the possible ways sensory constellations actually function and how these create thoroughly different but rich representations of the physical (Fluegge, 2003).

As there cannot be a single way of creating knowledge, only such a broad, socioculturally-founded yet materially based perspective will perhaps allow us to fill in the missing elements.

## Author Contributions

NAH conceptualized the present work and wrote the current version for publication.

## Conflict of Interest

The author declares that the research was conducted in the absence of any commercial or financial relationships that could be construed as a potential conflict of interest.
